# A Chemogenomic Screen Reveals Novel Snf1p/AMPK Independent Regulators of Acetyl-CoA Carboxylase

**DOI:** 10.1371/journal.pone.0169682

**Published:** 2017-01-11

**Authors:** Bruno L. Bozaquel-Morais, Juliana B. Madeira, Thiago M. Venâncio, Thiago Pacheco-Rosa, Claudio A. Masuda, Monica Montero-Lomeli

**Affiliations:** 1 Instituto de Bioquímica Médica Leopoldo de Meis, Programa de Biologia Molecular e Biotecnologia, Universidade Federal do Rio de Janeiro, Rio de Janeiro, Brazil; 2 Universidade Estadual do Norte Fluminense Darcy Ribeiro, Campos dos Goytacazes, Rio de Janeiro, Brazil; Texas A&M University College Station, UNITED STATES

## Abstract

Acetyl-CoA carboxylase (Acc1p) is a key enzyme in fatty acid biosynthesis and is essential for cell viability. To discover new regulators of its activity, we screened a *Saccharomyces cerevisiae* deletion library for increased sensitivity to soraphen A, a potent Acc1p inhibitor. The hits identified in the screen (118 hits) were filtered using a chemical-phenotype map to exclude those associated with pleiotropic drug resistance. This enabled the identification of 82 ORFs that are genetic interactors of Acc1p. The main functional clusters represented by these hits were “transcriptional regulation”, “protein post-translational modifications” and “lipid metabolism”. Further investigation of the “transcriptional regulation” cluster revealed that soraphen A sensitivity is poorly correlated with *ACC1* transcript levels. We also studied the three top unknown ORFs that affected soraphen A sensitivity: *SOR1* (*YDL129W*), *SOR2* (*YIL092W*) and *SOR3* (*YJR039W*). Since the C18/C16 ratio of lipid acyl lengths reflects Acc1p activity levels, we evaluated this ratio in the three mutants. Deletion of *SOR2* and *SOR3* led to reduced acyl lengths, suggesting that Acc1p is indeed down-regulated in these strains. Also, these mutants showed no differences in Snf1p/AMPK activation status and deletion of *SNF1* in these backgrounds did not revert soraphen A sensitivity completely. Furthermore, plasmid maintenance was reduced in *sor2Δ* strain and this trait was shared with 18 other soraphen A sensitive hits. In summary, our screen uncovered novel Acc1p Snf1p/AMPK-independent regulators.

## Introduction

Cytosolic acetyl-CoA carboxylase (ACCase) plays a key role in lipid metabolism. This enzyme catalyses the carboxylation of acetyl-CoA, producing malonyl-CoA, a critical intermediate in fatty acid biosynthesis. In *Saccharomyces cerevisiae*, two ACCase isoforms were found, Acc1p, located in the cytoplasm, and Hfa1p, located in the mitochondrial matrix and less studied. Acc1p is essential in all organisms Even the supplementation of growth medium with long-chain fatty acids does not reverse the lethality of its deletion in yeast [1]. This suggests that Acc1p plays additional biochemical roles, other than involvement in fatty acid synthesis. In fact, malonyl-CoA produced by ACC1p is used not only by the fatty acid synthase to produce medium-chain fatty acids but is also a substrate for long-chain fatty acids required for the synthesis of sphingolipids and histone acetylation [2–4]. Understanding the regulation of Acc1p activity and the consequences of its inhibition is essential since the protein is a potential target for the treatment of metabolic syndrome, obesity, cancer and microbial infections, among others [5–7].

Yeast Acc1p is encoded by the *ACC1* gene (*YNR016C*) and shares 63% similarity with human acetyl-CoA carboxylase (ACC1). Both are regulated by phosphorylation in response to the energetic status of the cell. The kinase responsible for the human acetyl-CoA carboxylase phosphorylation is the AMP-activated protein kinase (AMPK) which in yeast is named Snf1p [8,9]. Snf1p/AMPK might not be the only kinase that regulates acetyl-CoA carboxylase activity, as in yeast, 30% of the Acc1p pool is phosphorylated in a *snf1Δ* background [10]. Protein phosphatase responsible for Acc1p dephosphorylation and subsequent activation remains unknown, although studies in mammalian and yeast models suggest that a PP2A-like protein phosphatase may be involved [11]. In contrast to the mammalian enzyme, the yeast Acc1p is not regulated allosterically by citrate [3]. Yeast *ACC1* is also transcriptionally activated by the Ino2p/Ino4p transcription factors and repressed by Opi1p [12]. This transcriptional regulation appears to be important for cellular response to the availability of metabolites inositol and choline [1].

Different high-throughput approaches have been used for studying the regulation of proteins and protein-protein interactions. Yeast is one of the more popular systems used because of the high amount of genetic tools available. We decided to use a chemogenomic approach, with the aim of identifying new Acc1p interactors. Chemogenomic screens were originally designed as a strategy to identify specific drug targets. In these screens, an organism or cells are challenged with a library of chemicals and the genomic or proteomic responses are studied [13–18]. Here, we used a single drug to screen a gene deletion library of *S*. *cerevisiae*. From the known Acc1p inhibitors, we selected the drug soraphen A [10], a macrocyclic polyketide originally isolated from the myxobacterium *Sorangium cellulosum* [19]. Soraphen A is highly specific for Acc1p, with no other target reported. It allosterically inhibits Acc1p activity by binding to the biotin carboxylase (BC) domain with a high affinity (*K*_d_ of ~1 nM), impairing oligomerisation and full activation of Acc1p [20,21]. Since Acc1p activity is essential for growth, soraphen A has served as a tool to indirectly measure the enzyme’s activity [10,22,23]: mutant strains with low Acc1p activity are more drug sensitive than strains with higher Acc1p activity. This approach can reveal several features, such as novel Acc1p regulators, novel biochemical buffering mechanisms triggered by Acc1p inhibition and novel roles of Acc1p in cell physiology. We identified 118 ORFs that affect soraphen A sensitivity in yeast. Among these, 82 ORFs were primarily related to Acc1p activity, i.e. ORFs that are potential new regulators of Acc1p or participates in processes associated with Acc1p functions that are not yet fully understood. Detailed investigation of three ORFs with unknown functions revealed novel Snf1p-independent regulatory processes affecting Acc1p activity. Unexpectedly, one of them, *SOR2*, links genomic stability to Acc1p activity.

## Results

### Chemogenomic screen for soraphen A hypersensitivity

**Fig 1:**
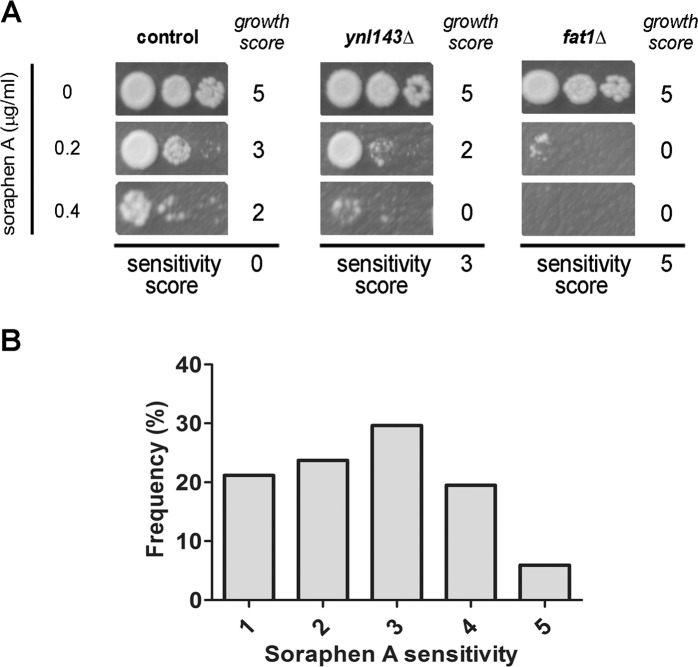
Confirmation of preliminary hits. (A) Following soraphen A screen, mutant strains considered to be sensitive were confirmed using a multi-spot semi-quantitative assay. Serial dilutions of pre-cultures of each mutant were spotted onto YPD agar containing the indicated concentrations of soraphen A. Growth on each plate was scored 0 to 5. The sensitivity score was calculated as the sum of all scores in the presence of soraphen subtracted from the score obtained in the absence of soraphen. Examples of three different strains are shown. Sensitivity score for each strain is presented in S2 Table (as a mean of four independent experiments). The *lys2Δ* strain was employed as a control. (B) Distribution of sensitivity scores. One-hundred and seventeen strains were confirmed to be soraphen A sensitive. Sensitivity scores ranged from 1 to 5 (compared with the control strain, which was considered “0”).

Using the previously reported IC_50_ value for a wild-type (WT, BY4741) *S*. *cerevisiae* strain (0.19 μg/ml) [22], we tested the growth of ~4860 null mutants in rich medium (YPD) containing 0.2 μg/ml or 0.4 μg/ml of soraphen A. After 72 h at 30°C, yeast growth was registered and scored (the scoring method is described in the Materials and Methods section). The growth of 156 mutants was inhibited, compared with no-drug YPD controls (S1 Table). To verify these preliminary hits, we rescreened the mutants under the same growth conditions, but using a multi-spot assay, where each strain was plated at three different cellular dilutions. This allowed us to assess their sensitivity to soraphen A on a semi-quantitative scale (Fig 1A). Using this assay, 127 hits were confirmed (S2 Table), and nine strains with deleted dubious ORFs were excluded from the dataset at this point. According to the soraphen A sensitivity score, these hits were distributed from 1 to 5, and 55% of 118 confirmed hits showed moderate to high sensitivity (score from 3 to 5) (Fig 1B and S2 Table).

To verify the reliability of the data, we searched for previously reported soraphen A sensitive strains within the literature. We successfully identified four such sensitive strains: *sit4Δ*, *sap190Δ*, *sur4Δ* and *fen1Δ* [3,22,24]. We also observed some resistant mutants (data not shown), including *snf1Δ* and *snf4Δ*. During the course of our work, results of a large automated screening of heterozygous and homozygous *S*. *cerevisiae* deletion collections encompassing ~1800 compounds, including soraphen A, were published [25]. Although the results of soraphen A screen were not further explored by the authors, this work identified 86 hypersensitive mutants The published approach by Hoepfner et al. is different from ours. First, the published study employed a diploid and not a haploid collection, including both homozygous and heterozygous mutants, which allowed the investigation of essential genes excluded from our collection. Second, the authors performed the screen in liquid medium using pools of mutants. Finally, soraphen A sensitivity was assessed by RT-PCR (as each mutant in the library had a unique identifying sequence code). Despite these differences, we identified 21 common hits (S2 Table, labelled as “coincident genes”), which represented 26% of our original hits. Altogether, we conclude that our data are reliable.

**Fig 2:**
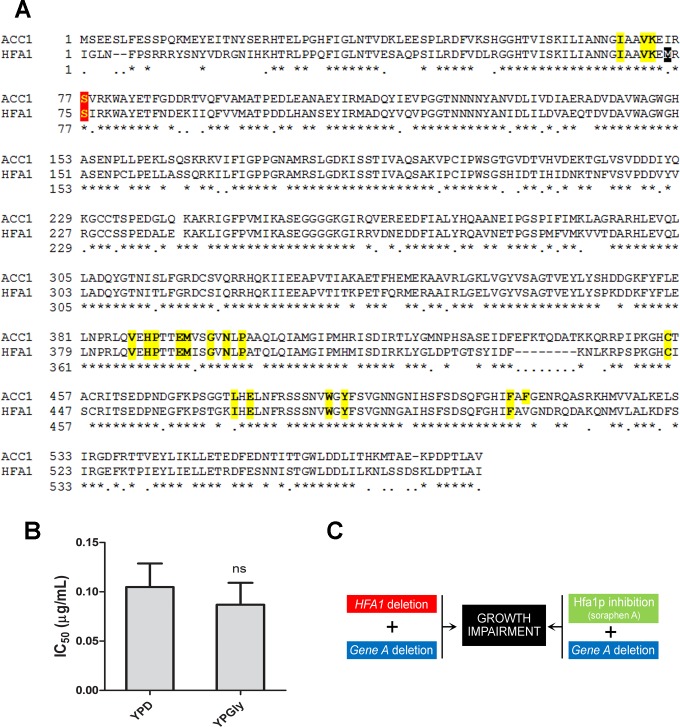
Screening is not biased by Hfa1p. (A) Alignment of Hfa1p and Acc1p biotin carboxylase (BC) domain sequences. Soraphen A binds to the BC domain of Acc1p blocking its oligomerization [20]. The residues (18) that were previously shown to interact with soraphen A in Acc1p BC domain [20] are marked in yellow. The regulatory serine-77 is marked in red. Note that Hfa1p translation begins at a non-canonical (Ile) start codon in position -71 relative to the annotated initial methionine, marked in black [26,27]. (B) Soraphen A sensitivity in fermentative and respiratory substrates. WT strain was grown for 48 h in liquid YP medium containing 2% glucose (YPD) or 2% glycerol (YPGly) in the presence of increasing concentrations of soraphen A. Soraphen A IC_50_ values were determined as described in Methods section. Results are the means of four independent experiments ± standard deviations (non-paired t-test; ns, non-significant).(C) The screen does not select negative interactors of *HFA1*. *hfa1Δ* strain shows no growth defects when grown under standard conditions in YPD; however, cell growth is impaired upon deletion of gene *A*, a negative interactor. If Hfa1p would be inhibited by soraphen A, a strain with a deletion of gene *A* (or any negative interactor of *HFA1*) would be more sensitive to the drug. That was not the case as only 3 out of 50 negative interactors were identified in the screen (*CPT1*, *PBS2* and *TPS2*), however they were filtered in subsequent analysis.

As our main goal was to identify Acc1p interactors, we addressed if our results could be biased by the inhibition of a second acetyl-CoA carboxylase, Hfa1p, localized in the mitochondrial matrix. To the best of our knowledge, there is not notion in the literature whether Hfa1P is also a soraphen A target. Hfa1p and Acc1p are thought to have evolved from a single gene [26]. Soraphen A interacts with 18 amino acids in the BC domain [20]. To investigate whether soraphen A binds to Hfa1p, BC domains of both isoforms were aligned (Fig 2A). We discovered 73% identity and 84% positive substitutions between the sequences. Furthermore, from the soraphen A-interacting residues, only residue F512 was not conserved between Hfa1p and Acc1p. This suggested that both enzymes could potentially be inhibited by soraphen A. To address if the possible inhibition of Hfa1p by soraphen A could interfere with our results, first we sought for *hfa1Δ* strain among our hits, this results was further confirmed using a multi-spot semi-quantitative assay. Afterwards we tested the drug’s effect on growth of WT strain on glycerol, a respiratory substrate. Hfa1p is essential for respiration by providing substrates for the mitochondrial type II fatty acid biosynthetic pathway (FAS II). If both ACC1p and Hfa1p isoforms contributed to sensitivity we expected a higher IC_50_ for soraphen A in glycerol but as shown in Fig 2B, the IC_50_ for soraphen A was not significantly different regardless of the carbon source used. Since Hfa1p provides substrates for FAS II, its activation in cells treated with soraphen A could alleviate inhibition of Hfa1p. FAS II is a multimeric complex and the loss of any of its subunits impairs mitochondrial fatty acid synthesis. We therefore checked whether deletion of some of these subunits (Cem1p, Oar1p, Htd2p and Etr1p) affected soraphen A sensitivity, but none of the mutant strains were more sensitive to the drug than WT (S1A Fig). Finally,we searched for negative *HFA1* interactors among our screen hits, i.e. genes whose deletion in an *hfa1Δ* background led to reduced growth under standard conditions (Fig 2C). The screen poorly identified *HFA1* negative interactors. Out of 50 *HFA1* negative interactors reported thus far, just three of them, *CPT1*, *PBS2* and *TPS2*, were identified in our screen; only *TPS2* was retained after we filtered our data with a chemical-phenotype map (vide infra). Also the results of the screening of Hoefner et al., did not found any *HFA1* interactor. Under the conditions tested, the evidences point out that the possible inhibition of Hfa1p does not interfere with our results. Therefore we considered that our hits refer to Acc1p regulation.

### Chemical-phenotype analysis reveals 82 novel putative regulators of Acc1p activity

**Fig 3:**
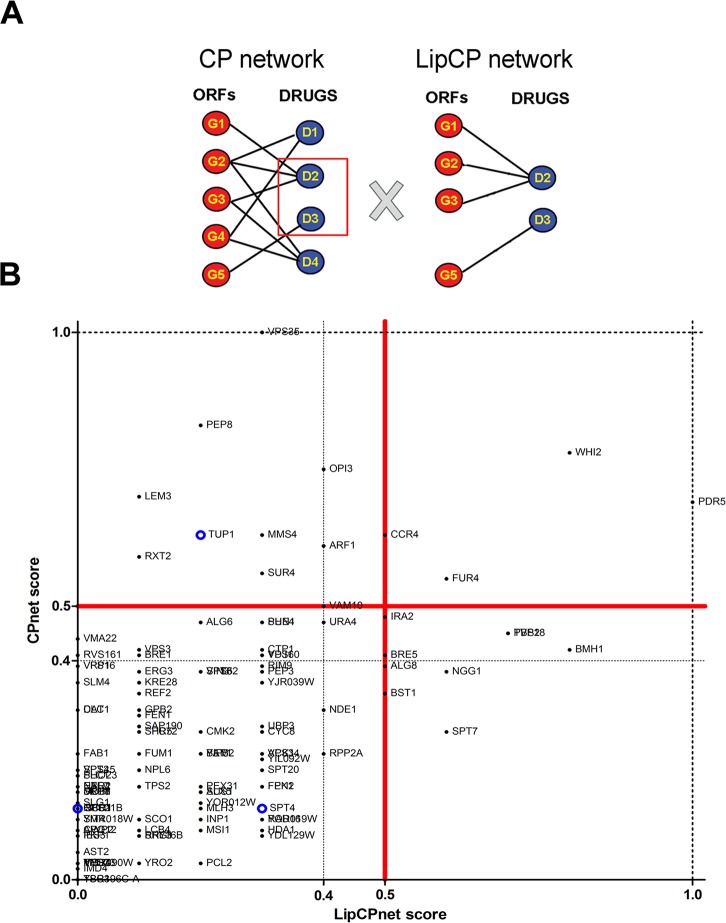
Chemical phenotype (CP) analysis identifies hits that according to rank plot analysis are specifically associated with soraphen A treatment. (A) CP network is a bipartite hybrid network comprising ORFs and drugs [15]. An ORF is connected to a drug when its deletion increases sensitivity to that drug. LipCP network was extracted by choosing drugs that target the lipid metabolism (S3 Table) and their connected ORFs from the CP network (S4 Table). (B) ORFs identified among our final hits (S2 Table), plotted according to their relative connectivity in both CP and LipCP networks. The score represents the number of drug connections of an ORF in each network, normalised so that the most connected ORF scores 1 (S5 Table). Quadrants are numbered as shown. ORFs that are located in the third quadrant and with degree lower than 0.4 in both networks, represented by dotted lines, were considered to be primarily associated with soraphen A treatment. Final hits are marked as black dots. Blue circles represent ORFs that were missed by the screen but were added in later after individual re-testing (*SPT4*, *SFP1* and *TUP1*; please refer to the text).

We considered as ACC1p interactors ORFs that fit at least one of the following three possibilities: i) ORFs whose deletion constitutively reduce Acc1p activity, ii) ORFs that are important upon inhibition of Acc1p; or iii) ORFs whose deletion increases cellular demand for Acc1p activity. To identify these interactors, we applied a more stringent filter to the 118 confirmed hits, to exclude genes associated with non-specific responses to drugs, such as drug extrusion or detoxifying systems. We also excluded strains where soraphen A sensitivity was potentially linked to a general inhibition of lipid metabolism. We took advantage of a previously published chemical-phenotype network (CP network), which is a bipartite hybrid network comprising 426 chemicals and 5233 ORFs whose deletion leads to sensitivity to those compounds [15]. Using these data, we constructed a subnetwork, the Lipid Chemical Phenotype (LipCP) network (S3 Table), consisting only of drugs that are known to affect lipid metabolism (e.g. imidazoles, triazoles and polyenes; S4 Table) (Fig 3A). Then, we evaluated the relative connectivity of our hits in both CP and LipCP networks using a rank plot analysis [28] (the connectivity scores obtained in each network are presented in S5 Table). The rank plot presents ORFs in four quadrants, highlighting their relative importance (based on degrees) in either network (Fig 3B).

We assumed that the Acc1p interactors must show low connectivity in both CP and LipCP networks, and would be localised in the third quadrant of the rank plot. We selected the appropriate hits and adopted yet another, even more stringent filter; we arbitrarily discarded hits with scores higher than 0.4 in both the CP and LipCP networks. We thus obtained a final group of 82 Acc1p interactors (Table 1). Strikingly, 40% (37) of the ORFs were not present in the LipCP network (score 0), which suggested that indeed they were highly specific to Acc1p inhibition.

**Table 1:** Candidate Acc1p interactors with their respective soraphen A sensitivity scores.

*SYSTEMATIC*	*NAME*	SCORE	*SYSTEMATIC*	*NAME*	SCORE
YNL097C*	*PHO23*	5	*YCR028C*	*FEN2*	4
YDL047W*	*SIT4*	5	*YKR028W*	*SAP190*	4
YBR058C-A*	*TSC3*	5	*YER151C*	*UBP3*	4
YDR294C*	*DPL1*	4	*YDR484W*	*VPS52*	4
YMR100W*	*MUB1*	4	*YBR112C*	*CYC8*	3
YDL052C*	*SLC1*	4	*YNR047W*	*FPK1*	3
YER161C*	*SPT2*	4	*YPL262W*	*FUM1*	3
YAL016W*	*TPD3*	4	*YAL056W*	*GPB2*	3
YLR024C*	*UBR2*	4	*YOR171C*	*LCB4*	3
YPL045W*	*VPS16*	4	*YPL164C*	*MLH3*	3
YGL104C*	*VPS73*	4	*YMR091C*	*NPL6*	3
YIL040W*	*APQ12*	3	*YLR148W*	*PEP3*	3
YGR167W*	*CLC1*	3	*YGR004W*	*PEX31*	3
YAL013W*	*DEP1*	3	*YOL110W*	*SHR5*	3
YFR019W*	*FAB1*	3	*YOL148C*	*SPT20*	3
YOL112W*	*MSB4*	3	*YDL129W*		3
YJL136C*	*RPS21B*	3	*YJR039W*		3
YGL095C*	*VPS45*	3	*YDL203C*	*ACK1*	2
YER101C*	*AST2*	2	*YOR011W*	*AUS1*	2
YGL206C*	*CHC1*	2	*YOL016C*	*CMK2*	2
YKL096W-A*	*CWP2*	2	*YDR532C*	*KRE28*	2
YML113W*	*DAT1*	2	*YDL127W*	*PCL2*	2
YIL042C*	*PKP1*	2	*YBR114W*	*RAD16*	2
YLR337C*	*VRP1*	2	*YBR037C*	*SCO1*	2
YBR196C-A*		2	*YOL004W*	*SIN3*	2
YMR090W*		2	*YDL013W*	*SLX5*	2
YNL136W*	*EAF7*	1	*YAL047C*	*SPC72*	2
YLR052W*	*IES3*	1	*YLR055C*	*SPT8*	2
YML056C*	*IMD4*	1	*YLR240W*	*VPS34*	2
YPR031W*	*NTO1*	1	*YGL161C*	*YIP5*	2
YGL038C*	*OCH1*	1	*YNL021W*	*HDA1*	1
YOR008C*	*SLG1*	1	*YMR204C*	*INP1*	1
YBR077C*	*SLM4*	1	*YBR195C*	*MSI1*	1
YCR081W*	*SRB8*	1	*YJL078C*	*PRY3*	1
YMR018W*		1	*YDR195W*	*REF2*	1
*YBR041W*	*FAT1*	5	*YMR063W*	*RIM9*	1
*YCR034W*	*FEN1*	5	*YNL069C*	*RPL16B*	1
*YDR074W*	*TPS2*	5	*YBR258C*	*SHG1*	1
*YIL092W*		5	*YBR054W*	*YRO2*	1
*YER155C*	*BEM2*	4	*YOR012W*		1
*YLR056W*	*ERG3*	4	*YOR019W*		1

* ORFs that have not been linked with drugs that affect lipid metabolism before (degree 0 in the LipCP network).

ORFs with high scores in both networks (second quadrant) are systematically identified in chemogenomic screens. They are associated with general drug response mechanisms, such as *PDR5*, the main ABC transporter responsible for soraphen A extrusion [29], and *WHI2*, involved in general stress response. ORFs poorly connected to general drugs (low score in CP network) but scoring high in the LipCP network (fourth quadrant) were probably identified in our screen because they could be involved in mechanisms that globally affect lipid metabolism. Thus, the effect of their deletion on soraphen A sensitivity might not be restricted to Acc1p activity regulation. For example, *IRA2* from the Ras-cAMP signalling pathway was previously thought to participate in the regulation of carbon fluxes into storage lipids [30], which might affect lipid metabolism in general. Thus, we are confident that the hits in these remaining quadrants are not Acc1p modulators.

### Functional analysis of novel Acc1p regulators

**Fig 4:**
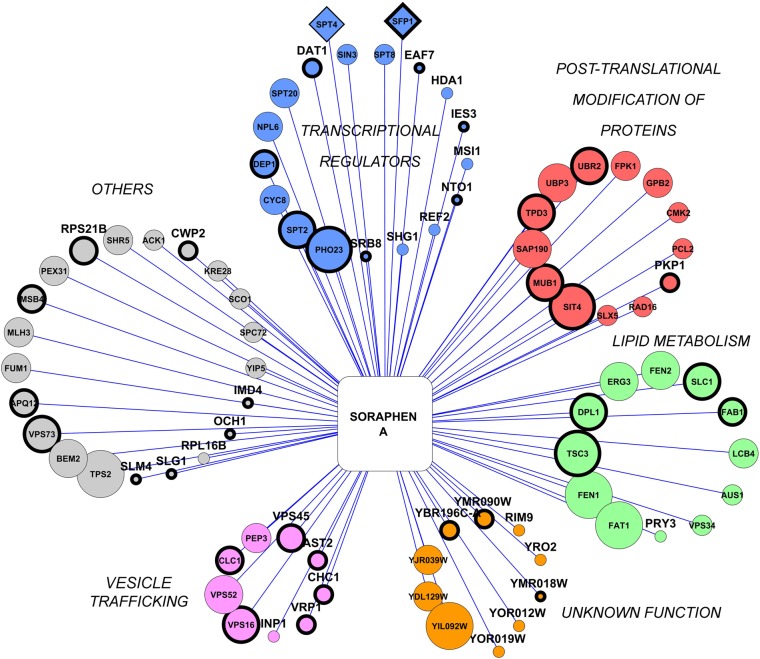
Eighty-four ORFs are strongly linked to Acc1p inhibition. Final hits (S2 Table) were filtered by rank plot analysis employing the chemical phenotype network (Fig 3). The ORFs were manually clustered according to their description in Saccharomyces Genome Database (SGD, www.yeastgenome.org). Node size represents the inhibition of growth score in the presence of soraphen A (from 5 to 1). Diamond-shaped nodes represent ORFs that were missed by the screen but added in later after individual re-testing (Fig 5). In these cases, no sensitivity score was assigned. Thicker nodes represent ORFs that have not been previously associated with lipid metabolism inhibition (LipCP network score equal to zero).

The final Acc1p interactors (82) were clustered according to their physiological roles (Fig 4). The major groups were “*transcriptional regulators*”, “*post-translational modification of proteins*”, “*lipid metabolism*”, “*ORFs with unknown function*” and “*vesicle trafficking*”. Because of the considerable amount of information generated by our screen, we decided to focus on “*transcriptional regulators*” and “ORFs with unknown function” clusters.

**Fig 5:**
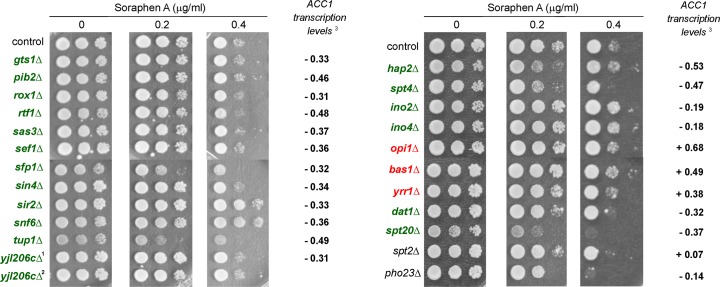
*ACC1* transcriptional levels do not correlate with soraphen A sensitivity. Mutant strains with deletion of genes encoding transcriptional factors (TFs) that affect *ACC1* transcript levels were retested for soraphen A sensitivity as before (Fig 3). Published *ACC1* transcript levels in these mutants [32] are shown. TFs mutant strains are marked in green or red, depending on whether *ACC1* transcription was down-regulated or up-regulated, respectively [31]. TF mutant strains identified by our screen but with unaltered *ACC1* transcription [30] are marked in black. The *lys2Δ* strain was employed as a control. The results are representative of four independent experiments. ^1, 2^ Two *yjl206cΔ* strains were present in our collection and both were tested.

The transcriptional cluster contained 17 genes. Interestingly, *INO2* and *INO4* were not identified in our screen nor in the screen by Hoepfner et al. (2014), even though their deletion down-regulates *ACC1* transcription levels [1]. Surprisingly, a more detailed analysis revealed that out of 20 transcription factors (TFs) reported to affect *ACC1* transcription [31,32], just two, *DAT1* and *SPT20*, were identified in our screen and none of them was identified by Hoepfner et al. (2014). Because of the high discrepancy between literature and our results, we re-tested all 20 TFs plus TF-deleted strains found only in our screen (*spt2Δ* and *pho23Δ*) (Fig 5) and compared their soraphen A sensitivities with the reported levels of *ACC1* transcription [32]. Indeed, our screen missed just 3 of 20 re-tested strains (*SFP1*, *TUP1* and *SPT4*) which suggests that soraphen A sensitivity and *ACC1* transcription are poorly correlated, These three missing mutants were then submitted to the same chemical-phenotype analysis as before (Fig 3B, blue circles), and *SFP1* and *SPT4* were included in our final set of candidate ORFs (Fig 4, diamond-shaped nodes). In conclusion, six hits (*sfp1Δ*, *spt4Δ*, *dat1Δ*, *spt20Δ*, *spt2Δ* and *pho23Δ*) are good TF candidates for further studies focused on transcriptional regulation of *ACC1*.

### Three ORFs with unknown function affect Acc1p activity

**Fig 6:**
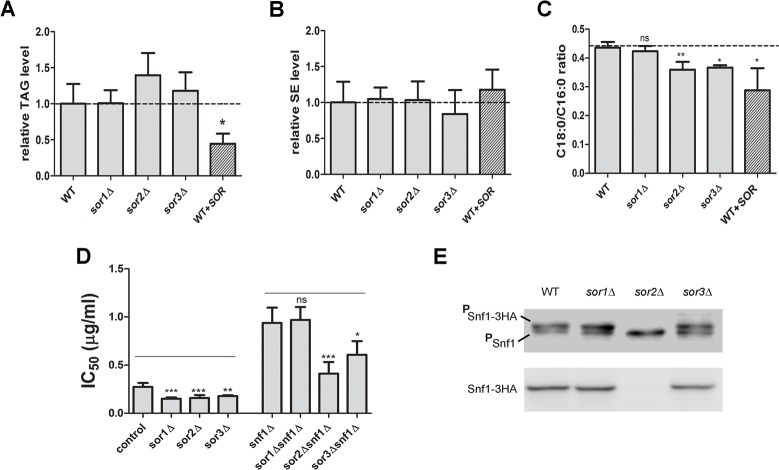
Characterization of neutral lipids, acyl lengths and soraphen A sensitivity of *sor1Δ*, *sor2Δ* and *sor3Δ* mutants. For lipid analysis, *SOR* null mutants and WT (BY4741) strains were grown in YPD. WT was also grown in the presence of a sub-lethal concentration of soraphen A (0.1 μg/ml). After 24 h, the total lipids were extracted and the lipid profile was determined by TLC (A,B) or GC/MS (C). Triacylglycerol (A) and sterol ester (B) contents were estimated from TLC plates by densitometry and were normalised to the values obtained for WT grown in YPD. (C) C18:0 and C16:0 extracted from total lipids were quantified and the C18:0/C16:0 ratio of each strain is shown. Results presented in (A), (B) and (C) are means of three independent experiments ± standard deviations (non-paired t-test; **, P < 0.01; *, P < 0.05; ns, non-significant). (D) Soraphen IC_50_ values for single and *sorXΔsnf1Δ* mutants were determined as described in Methods section. As controls, *lys2Δ* and *snf1Δ* strains were employed. Results are the means of at least four independent experiments ± standard deviations (non-paired t-test; **, P < 0.01; *, P < 0.05; ns, non-significant). (E) Strains transformed with pSNF1 (pYC::SNF1-3HA) were grown in liquid YPD medium to log-phase and proteins were extracted for western blot analysis. Blots were probed with anti-phospho AMPK antibody (upper panel) or anti-hemagglutinin antibody (lower panel). HA-tagged Snf1p and endogenous Snf1p in phosphorylated form are designated as ^P^Snf1-3HA and ^P^Snf1, respectively. Total tagged Snf1p is designated as Snf1-3HA.

The main challenge following genome sequencing comprises the characterization of proteins with unknown function that are often divergent or lineage-specific. In the present study, we focused on the top three most-sensitive candidates with unknown function: *YDL129W*, *YIL092W* and *YJR039W*, which we called *SOR1*, *SOR2* and *SOR3*, respectively, as in “SORaphen sensitive mutants”. *SOR1*, *SOR2* and *SOR3* encode proteins with molecular weights of 32.92 kDa, 71.02 kDa and 127.42 kDa, respectively. All of them were found to be unique to fungi, as verified by BLAST (data not shown). To test whether these hits were direct Acc1p modulators, we evaluated their triacylglycerol and steryl ester levels, and compared these to levels in soraphen A-treated cells. Treatment with a sub-lethal concentration of soraphen A (0.1 μg/ml) reduced the triacylglycerol, but not steryl ester, levels in WT cells (Fig 6A and 6B). Interestingly, in *sor1Δ sor2Δ* and *sor3Δ*strains, no significant differences in triacylglycerol or steryl ester levels were observed. This result was unanticipated; however, we next investigated the length of the acyl groups in the total cellular lipid fractions by GC/MS (Fig 6C, Table 2), as it has been recently shown that such parameter is affected by the activity of Acc1p [10,33]. Indeed, inhibition of Acc1p by soraphen A reduced the C18/C16 ratio by ~40% in WT cells. We also observed a reduction in the C18/C16 ratio in *sor2Δ* and *sor3*Δ cells (~20% in both) but not in *sor1*Δ cells. Taken together, these results suggested Sor2p and Sor3p as probable Acc1p regulators.

**Table 2:** Acyl composition in total lipid extracts of WT and *SOR* null mutants after 24 h growth in rich YPD medium, as determined by GCMS.

Acyl	WT	*sor1Δ*	*sor2Δ*	*sor3Δ*	WT+SOR
C18:0	7.78 ± 2.17	7.25 ± 0.37	6.31 ± 0.70	6.28 ± 1.12	4.34 ± 4.84
C16:0	18.89 ± 4.67	17.47 ± 1.59	16.41 ± 0.68	16.96 ± 3.23	6.39 ± 2.96
C18/C16	0.44 ± 0.011	0.423 ± 0.011	0.360 ± 0.016	0.367 ± 0.005	0.29 ± 0.044

WT+SOR, wild type strain grown under the same conditions in the presence of soraphen A (0.1 μg•ml^-1^). Fatty acid content is expressed as μg per 10^8^ cells. Values are means and standard deviations from three independent experiments.

The Snf1p/AMPK pathway is the most studied Acc1p regulatory pathway. Therefore, we investigated whether Sor2p and Sor3p regulate Acc1p activity via that pathway or whether they participate in novel regulatory pathways. We deleted each *SOR* gene in a *snf1Δ* background, which is hyper-resistant to soraphen A (IC_50_ = 0.95 μg/ml) when compared with WT (IC_50_ = 0.19 μg/ml) [22,23] Soraphen A sensitivity of *sor1Δ snf1Δ* mutant was unaltered in relation to *snf1Δ*, while in *sor2Δ snf1Δ* and *sor3Δ snf1Δ*, it was ~50% and ~30% lower than that measured for *snf1Δ* cells (Fig 6D). These results indicated that *SOR2* and *SOR3* act independently of Snf1p. Furthermore, we found no difference between Snf1p phosphorylation levels in *SOR* knockout mutants (Fig 6E), which suggested that the Snf1p/AMPK pathway was not compromised in these mutants. In these experiments, it was striking that *sor2Δ* was unable to retain a plasmid containing an epitope-tagged Snf1p. This phenotype was reproduced in independent clones, was exclusive to *SOR2* deletion and is further explored in the following section.

### Reduced plasmid maintenance in *sor2Δ* mutant

**Fig 7:**
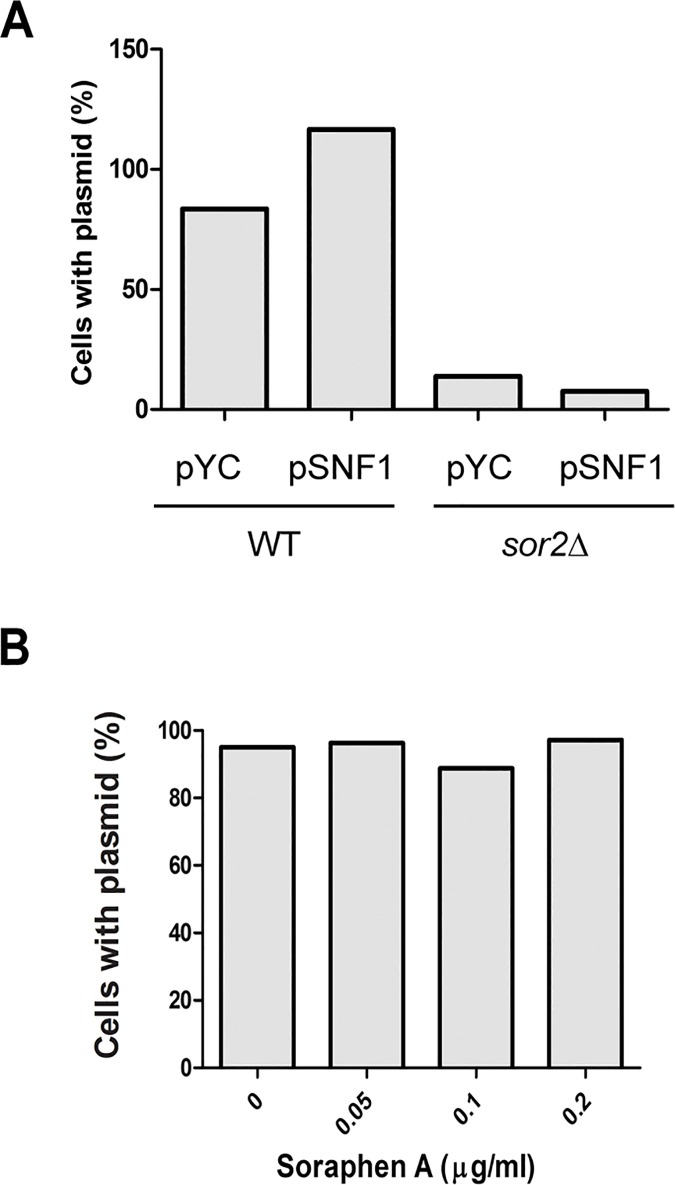
*SOR2* deletion reduced Acc1p activity and plasmid loss are not directly linked. (A) Strains transformed with pYC (empty vector) or pSNF1 (pYC::SNF1-3HA), both with *URA3* as a genetic marker, were grown for 48 h in liquid SD medium in the presence of selective pressure (without uracil supplementation). The percentage of plasmid-carrying cells was quantified by plating 200 cells of each culture on SC agar +ura or–ura, and colony counting after 3 da. For WT + pYC and WT + pYC:SNF1, n = 1; for *sor2Δ* + pYC and *sor2Δ* + pYC:SNF1, n = 3 (three different isolated clones were tested). (B) WT strain carrying pYC (empty vector) was grown for 48 h in liquid SD medium in the presence of selective pressure (without uracil), with the indicated concentrations of soraphen A. Plasmid stability was assessed as in (B) and the data are presented as the means from two experiments.

The failure of *sor2Δ* cells (Fig 6E) to maintain a plasmid encoding hemagglutinin-tagged Snf1p (Snf1p-HA), led us to investigate whether this was caused by *SNF1* overexpression. To test this, we also transformed WT and *sor2Δ* cells with an empty vector and compared the stability of both plasmids stabilities. The results revealed that, under selective pressure, *SNF1* overexpression did not affect plasmid maintenance in WT as both plasmids were maintained by more than 95% of the cells (Fig 7A). On the other hand, in *sor2Δ* strain, both plasmids were maintained in only ~10% of the population. We also tested whether the plasmids were lost as a consequence of reduced Acc1p activity (Fig 7B). We found that Acc1p inhibition by soraphen A did not induce plasmid loss in WT cells. We also tested if plasmid loss in *sor2Δ* strain was due to decreased repair of DNA, which could be a cause for genomic instability. However, *sor2Δ* was as sensitive as WT to the DNA damaging agents hydroxyurea (100mM) and methyl methanesulfonate (0.02%).

## Discussion

We screened a yeast deletion library to identify genes whose deletion conferred sensitivity to soraphen A, a potent Acc1p inhibitor, with the aim of finding new regulators or processes that are linked with Acc1p activity. The data generated were reliable as the screen identified all deletion strains previously reported as soraphen A sensitive. From the strains that were retested, we estimated a 10% rate of false negatives (data not shown). Chemogenomic screens are prone to identify mutants that affect drug sensitivity via unspecific mechanisms, as in the case of pleiotropic drug resistance. To overcome this issue, we employed a biosystematic approach. We successfully discarded 30% of the 118 initial hits, including *PDR5*, an ABC-transporter responsible for pumping soraphen A out of the cell [29].

By analysing the physiological functions of our final hits, we found that the major groups comprised “*transcriptional regulators*”, “*post-translational modification of proteins*”, “*lipid metabolism*”, “*ORFs with unknown function*” and “*vesicle trafficking*”. Further analysis of the “*transcriptional regulators*” cluster revealed that from 23 TFs previously reported as putative *ACC1* regulators [12,34,31,32], the deletion of only six of them correlated with altered soraphen A sensitivity. *ACC1* presents an upstream activation sequence recognised by the Ino2p/Ino4p complex and it was reported that *ACC1* transcription is up-regulated in an *opi1Δ* strain and down-regulated in *ino2Δ* and *ino4Δ* strains [1]; however, these strains did not present abnormal soraphen A sensitivity. This is in agreement with the poor correlation between mRNA and protein levels of many genes [35]. The final six TFs presented here (*SFP1*, *SPT4*, *DAT1*, *SPT20*, *SPT2* and *PHO23*) are promising targets for Acc1p modulation.

Together with *SIT4* and *SAP190*, in the “*post-translational modification of proteins*” cluster, we found putative regulatory elements for further study (Fig 5), such as catalytic or regulatory subunits of kinase complexes. None of these ORFs have been previously shown to interact physically or genetically with Acc1p and it would be interesting to investigate whether these kinases are directly involved in Acc1p phosphorylation. We also found five ORFs associated with ubiquitination (*UBP3*, *UBR2*, *MUB1*, *SLX5* and *RAD16*). There is evidence that the Ubp3p/Bre5p complex participates in Acc1p regulation: i), these two proteins interact physically with Acc1p [36]; and, ii), it has been demonstrated that acetyl-CoA carboxylase is subject of ubiquitin-mediated proteolysis in mammalian cells [37], although this phenomenon remains unclear in yeast.

A major concern that arose from our screen was that the mitochondrial acetyl-CoA carboxylase, Hfa1p, could also have been inhibited by soraphen A, as it shares with Acc1p all the residues previously shown to interact with soraphen A, except for F512. Our screen and the screen by Hoepfner et al. [25] failed to identify *HFA1* negative interactors. Also we observed that loss of any of the FASII subunits, that impair mitochondrial fatty acid synthesis as well as *HFA1* deletion, are not more sensitive to soraphen A. These results suggested that mitochondrial fatty acid synthesis does not play a role in the cellular response to soraphen A. The different conditions tested strongly indicate that the chemical-genetic interactions observed in this work are specific for Acc1p. However, to reach the conclusion that Hfa1p is not inhibited by soraphen A, this isoform should be purified and the enzymatic activity assayed in the presence of soraphen A.

From the ten ORFs with unknown functions, deletion of three of them resulted in a moderate to high sensitivity to soraphen A. These ORFs were named as *SOR1* (*YDL129W*), *SOR2* (*YIL092W*) and *SOR3* (*YJR039W*). To check whether these ORFs were constitutive Acc1p regulators, we measured the lengths of the acyl chains of cellular lipids in corresponding mutant strains. It has been shown that the reduction of mammalian acetyl-CoA carboxylase activity by soraphen A or its activation in yeast after a deletion of *SNF1* lead to shorter or longer acyl chains, respectively [10,33]. We observed reduced C18/C16 acyl ratios in *sor2Δ* and *sor3Δ* strains, as well as in WT treated with soraphen A. This indicated that Acc1p was constitutively low in these strains, suggesting that *SOR2* and *SOR3* are Acc1p regulators. The exact mechanisms by which Sor2p and Sor3p participate in Acc1p regulation remain elusive. However, it is clear that they do not involve indirect regulation via the modulation of Snf1p/AMPK activity (as already shown for *SIT4* and *SAP190*, for example [22], since Snf1p/AMPK phosphorylation levels were not altered in these strains and deletion of *SOR2* and *SOR3* in a *snf1Δ* background partially reversed soraphen A resistance.

We cannot make such conclusions regarding *SOR1*, however, as its deletion did not affect the C18/C16 ratio. However, it is important to mention that when grown on alternative carbon sources, such as glycerol, *sor1Δ* sensitivity was reversed, which was not the case of *sor2Δ* and *sor3Δ* (data not shown). We hypothesized that deletion of *SOR1* could either reduce cell fitness (which would increase its dependence on Acc1p activity) or impair any backup system responsible for sustaining Acc1p activity upon soraphen A treatment. Both hypotheses are still to be tested.

Finally, we observed that plasmid maintenance in *sor2Δ* strain was abnormal, which led us to hypothesize that genomic instability and Acc1p activity may be functionally related. Although plasmid/chromosome maintenance may be achieved by several different mechanisms, we observed a strong enrichment of mutants previously reported as genomic unstable among our hits. Interestingly, it has been previously shown that histone acetylation is associated with genomic stability [38] and increased H4K16 acetylation or loss of H4K16 deacetylase *SIR2* leads to genomic instability. Accordingly, *sor2Δ* could inhibit *ACC1* to increase the levels of acetyl CoA available for histone acetylation. However, soraphen A treatment of WT cells did not affect plasmid maintenance indicating that the problems in plasmid maintenance presented in the *sor2*Δ strain is not a consequence solely of the reduced ACC1p activity. On the other hand, it could be that the genomic instability could be the cause of the decrease in Acc1p activity. To test whether we could find a correlation between genes involved in genomic instability and the modulators of Acc1p, we re-analysed our screen primary hits. Among the 118 hits, we found 18 ORFs (15.3%, labelled as “*genomic instability*” in S2 Table) whose deletion affected plasmid/chromosome stability according to Saccharomyces Genome Database (SGD, www.yeastgenome.org) (as in August, 2016). This frequency of occurrence was higher than that observed in our knockout collection (279 out of 4860 strains, i.e. 5.7%); therefore, this ~2.5 fold enrichment was highly significant (P < 0.0001, hypergeometric distribution). As “genome instability” is a phenomenon that can be triggered by dysfunction of several processes it is difficult to determine which of these directly leads to *ACC1* down-regulation. Further work should be done to address this question.

## Materials and Methods

### Reagents

Soraphen A was a gift from Rolf Müller (Helmholtz-Zentrum für Infektionsforschung, Braunschweig, Germany) and was maintained as a 0.1 mg/ml stock solution in 10% methanol. All other reagents were from Sigma-Aldrich (St. Louis, MO, USA).

### Strains and media

*S*. *cerevisiae* strain BY4741 (MATa *his3Δ1 leu2Δ0 met15Δ0 ura3Δ0*) and yeast deletion library containing ~4860 strains where non-essential ORFs were individually replaced with a G418 (geneticin) resistance gene were obtained from Open Biosystems. The deletion collection was recovered from glycerol stocks via replica plating on rich YPD solid medium (1% yeast extract, 2% peptone, 2% glucose and 2% agar) at 30°C for 48 h. We verified the correct deletion of *YDL129W*, *YIL092W* and *YJR039W* genes by PCR and confirmed these to be the correct strains (data not shown). Double mutants *sor1Δ snf1Δ*, *sor2Δ snf1Δ* and *sor3Δ snf1Δ* were constructed by mating *sor1Δ*, *sor2Δ* or *sor3Δ* from the deletion library with a *snf1Δ* (MATα) strain and incubating in sporulation medium (0.02% raffinose, 0.3% potassium acetate) for 7 d. Double mutants were selected on glucose-MSG medium (2% glucose, 0.1% monosodium glutamate, 0.67% yeast nitrogen base without amino acids or ammonium sulphate) containing nourseothricin (100 μg/ml and geneticin (200 μg/ml), supplemented with histidine, leucine, methionine and uracil (0.003% final concentration of each). Isolated colonies were randomly picked and the presence of *SOR* and *SNF1* loci confirmed by PCR. Plasmid maintenance tests employed *sor2Δ* and WT transformed with pYC-SNF1:3HA plasmid, derived from pYC210, following the lithium acetate transformation method [39]. As means of selective pressure in these tests, uracil was removed from SD medium.

### Screening and scoring

Strains were spotted in triplicate with a 96-pin replicator onto a new YPD plate in the absence (control plate) or presence of 0.2 μg/ml or 0.4 μg/ml of soraphen A, based on the IC_50_ value previously reported by our group [22]. Growth was registered after 72 h of incubation at 30°C, as all strains should have reached stationary phase by that time point. The screening was performed twice. To identify sensitive mutants, the growth of each strain in the presence of soraphen A was compared with its growth in the absence of soraphen A, and an arbitrary score was assigned. The scores ranged from 5 to 0: a score of 0 reflected no difference in growth on drug-containing and control plates; a score of 5 reflected no growth in the presence of soraphen A. The final score was the sum of the scores obtained in the individual experiments. Strains with total scores equal to or higher than 5 were selected (S1 Table) and assessed in a multi-spot assay. These strains were grown in 96-well microplates filled with 0.2 ml of liquid YPD. Plates were incubated at 30°C under shaking for 48 h. From these pre-cultures, 10, 100 and 1000-fold dilutions were prepared in fresh 96-well microplates and were immediately spotted onto YPD solid medium supplemented with soraphen A with a replicator. Plates were incubated at 30°C. Growth was recorded after 48 h. Each plate received a score from 5 (full growth) to 0 (no growth). Final sensitivity score was calculated as the score on control YPD plate minus the sum of the scores obtained in the presence of soraphen A (some examples were shown in Fig 1A) (S2 Table).

### LipCP network

LipCP network is a subnetwork of the previously published CP network [15]. Briefly, the CP network consists of 426 chemical compounds with their chemical-genetic interactions in *S*. *cerevisiae* identified. To integrate these data with our results, we constructed a LipCP network by selecting drugs that are known to primarily affect lipid metabolic processes. We found 20 drugs that interfere with lipid biosynthesis/catabolism (e.g. cerulenin, azoles and peroxisome activators) and five sterol-binding compounds (e.g. polyenes, saponins) (S4 Table). Thus, the LipCP network encompassed these 25 drugs and their interactions with 3181 ORFs (edges and detailed information can be found in S3 Table).

### Network rendering and databases

The networks that are graphically represented in this paper were rendered with Cytoscape software [40]. Interaction data were obtained from BIOGRID [41]. Transcription factor information was obtained from YEASTRACT [42].

### Soraphen A sensitivity tests

For pre-cultures, cells were inoculated in liquid YPD and incubated for 24 h at 30°C with shaking. To confirm soraphen A sensitivity, OD_600_ was measured and pre-cultures were diluted in a microplate to obtain 10^7^, 10^6^ and 10^5^ cells/ml suspensions. Cellular suspensions were immediately spotted onto YPD solid medium supplemented and soraphen A (as indicated in the figures) with a replicator.

Plates were incubated at 30°C and pictures were taken after 2 d. For IC_50_ determinations, a 96-well microplate was filled with 100 μl/well of fresh YPD with increasing concentrations of soraphen A. Cellular suspensions were diluted to OD_600_ = 0.01 and 100 μl was added to each well (final concentrations of soraphen A ranged from 4 to 0.03 μg/ml). Plates were incubated at 30°C. Growth was recorded after 24 h by measuring absorbance at 600 nm in a plate reader (Spectramax 5M, Molecular Devices). The IC_50_ value was defined as the concentration of the drug required for 50% growth inhibition compared with the growth of drug-free controls. In both experiments, it was necessary to employ *lys2Δ* strain as the control since we observed that strains from the MATa deletion library were slightly more tolerant to soraphen A and other stressors than WT BY4741 [43].

### Lipid analysis

Yeast cells were grown for 24 h in YPD medium (soraphen A was added to 0.1 μg/ml final concentration, as indicated). Lipids were extracted and separated by thin-layer chromatography (TLC) as previously described [44,45]. Triolein, oleic acid and cholesteryl oleate were used as standards.

For acyl chain length determinations, 50 μg of tripentadecanoin, an internal standard, was added to cells prior homogenization. Total lipid extracts were dried under a stream of nitrogen and kept at -20°C until derivatization. For fatty acid derivatization, 500 μl of NaOH/methanol (20 g of NaOH in 1 L of methanol) was added to dried extracts and the tubes were incubated in boiling water for 15 min. After cooling, 500 μl of BF_3_-methanol 10% was added and the tubes were incubated as before. After cooling, 300 μl of NaCl-saturated aqueous solution was added. Finally, 300 μl of hexane was added to the tubes and, after homogenization and separation of phases, the organic phase was transferred to gas chromatography/mass spectrometry (GC/MS) glass vials.

Samples (2 μl) were injected into a DB-5ms column in a splitless mode using an autoinjector. Helium was used as a carrier gas. The inlets and MS source were maintained at 250°C. The oven temperature was maintained at 80°C for 4 min, then ramped to 170°C at a rate of 8°C/min and held for 10 min. The temperature was then ramped to 250°C at a rate of 4°C/min and held for 5 min.

### Snf1p/AMPK phoshorylation status

Yeast cells were grown for 48 h in YPD liquid medium and, then, inoculated in fresh YPD (initial O.D._600nm_ 0.25). Flasks were incubated under shaking (200 rpm) at 30°C and growth was followed till O.D._600nm_ ~1.0 (after 5 h). Culture samples were collected and total protein homogenates were prepared according to Yaffe and Schatz method [46] with minor modifications. NaOH and β-mercaptoethanol were added to a final concentration of 0.2 M and 1%, respectively. Tubes were incubated on ice for 2 minutes and, then, trichloroacetic acid was added to a final concentration of 6%. After incubation on ice for 10 minutes, tubes were centrifuged at maximum speed for 5 minutes. Supernatant was discarded and pellets were stored at -20°C until use or promptly resusspended in Laemlli sample buffer to obtain a 10-O.D. suspension. Proteins were separated by electrophoresis (SDS-PAGE, 10% SDS-polyacrylamide gel, 100 V, 2 hours) and electrotransferred to Immobilon-P for 20 min at 18 V in 25 mM Tris, 192 mM glycine and 10% methanol, using a trans-blot semi-dry cell (BioRad). Membranes were treated with anti-phospho-AMPKa Thr172 (Cell Signaling) or anti-HA (Sigma) antibodies. Blots were detected using the chemiluminescence ECL Plus kit (GE Healthcare).

### Plasmid maintenance test

For plasmid maintenance testing, *sor2Δ* or WT cells were transformed with pYC210 (empty vector) or pYC:SNF1-3HA plasmids and pre-grown under selective pressure in SD liquid medium without uracil (SD–ura) for 48 h at 30°C with shaking. Then, the selective pressure was removed by diluting the pre-cultures 100 times in SD liquid medium containing uracil (SD +ura) and the incubation was repeated. Pre-cultures (with selective pressure) and cultures (no selective pressure) were plated on both SD +ura (for total colony-forming units (CFU) assessment) and SD–ura (for plasmid-containing CFU assessment) solid media; 200 cells were plated for each condition and plates were incubated at 30°C. After 3 d, UFCs were manually counted. The percentage of plasmid-containing cells was calculated as the number of CFUs on SD–ura plate divided by the number of CFUs on SD +ura plate, and multiplied by 100.

## Supporting Information

S1 Fig Deletion of mitochondrial FASII subunits does not lead to altered sensitivity to soraphen A. Mutant strains with deletion of genes encoding FASII pathway enzymes were retested for soraphen A sensitivity as before (Fig 1). Soraphen A was added to the media at the indicated concentrations. Plates were incubated at 30°C for 3 d and growth was registered. The *lys2Δ* strain was employed as a control. The results are representative of three independent experiments.
(TIF)

S1 Table Sensitivity scores for null strains in the soraphen A screen, as described in the Material and Methods section. Only strains considered to be sensitive are listed.
(XLS)

S2 Table Final sensitivity scores for null strains after confirmation by multi-spot assay (as described in the Material and Methods section). Only confirmed strains are presented. “Coincident genes” refers to ORFs also identified in [25]. “Genomic instability” refers to ORFs identified as affecting plasmid/chromosome maintenance according to SGD.
(XLSX)

S3 Table Lipid Chemical Phenotype (LipCP) network edge list. (XLSX)

S4 Table Chemical compounds that affect lipid biosynthesis/catabolism extracted from Venancio et al. (2010) and included in the LipChemical phenotype network. (XLSX)

S5 Table Sensitivity scores for each identified ORF in both the original CP network and in LipCP network. Scores represent node degrees and were normalised based on the highest value of a node in each network. These results are visualised as a rank plot in Fig 4.
(XLSX)
