# Supplementary figures and images for: A Chemogenomic Screen Reveals Novel Snf1p/AMPK Independent Regulators of Acetyl-CoA Carboxylase
Source: PLoS One. 2017 Jan 11;12(1):e0169682. doi: 10.1371/journal.pone.0169682 (PMC5226726; doi:10.1371/journal.pone.0169682)

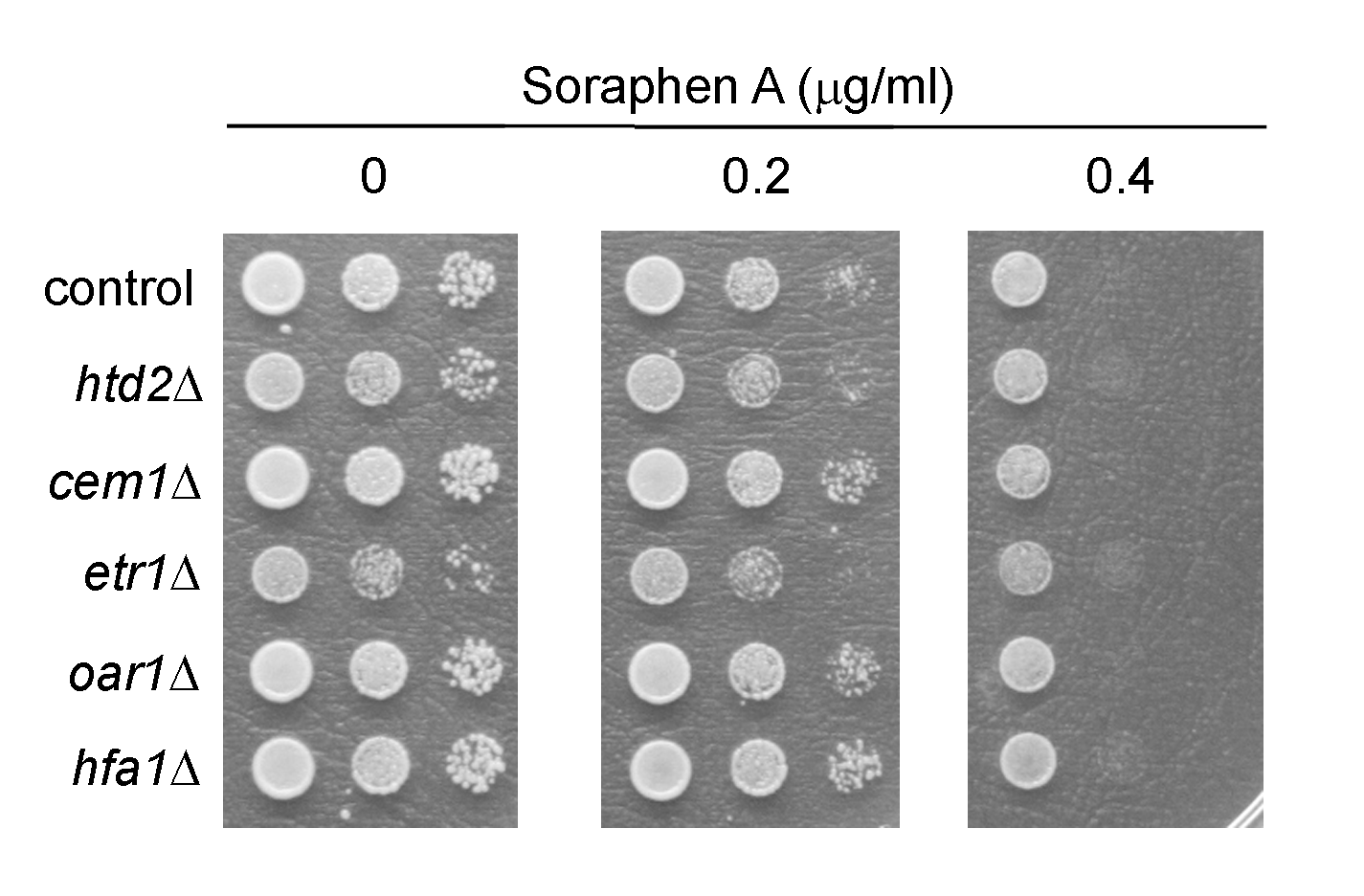

Supplement: S1 Fig — Mutant strains with deletion of genes encoding FASII pathway enzymes were retested for soraphen A sensitivity as before (Fig 1). Soraphen A was added to the media at the indicated concentrations. Plates were incubated at 30°C for 3 d and growth was registered. The lys2Δ strain was employed as a control. The results are representative of three independent experiments. (TIF) [file pone.0169682.s001.tif]
